# Reproducibility of automated habenula segmentation via deep learning in major depressive disorder and normal controls with 7 Tesla MRI

**DOI:** 10.1038/s41598-021-92952-z

**Published:** 2021-06-29

**Authors:** Sang-Heon Lim, Jihyun Yoon, Young Jae Kim, Chang-Ki Kang, Seo-Eun Cho, Kwang Gi Kim, Seung-Gul Kang

**Affiliations:** 1 Department of Health Sciences and Technology, Gachon Advanced Institute for Health Sciences and Technology (GAIHST), Gachon University, Seongnam-si, Republic of Korea; 2 Department of Biomedical Engineering, College of Medicine, Gachon University, Seongnam-si, Republic of Korea; 3 Department of Family Medicine, Yongin Severance Hospital, Yonsei University College of Medicine, Yongin, Republic of Korea; 4 Department of Radiological Science, College of Health Science, Gachon University, Incheon, Republic of Korea; 5 Department of Psychiatry, Gil Medical Center, Gachon University College of Medicine, Incheon, Republic of Korea

**Keywords:** Biomedical engineering, Depression

## Abstract

The habenula is one of the most important brain regions for investigating the etiology of psychiatric diseases such as major depressive disorder (MDD). However, the habenula is challenging to delineate with the naked human eye in brain imaging due to its low contrast and tiny size, and the manual segmentation results vary greatly depending on the observer. Therefore, there is a great need for automatic quantitative analytic methods of the habenula for psychiatric research purposes. Here we propose an automated segmentation and volume estimation method for the habenula in 7 Tesla magnetic resonance imaging based on a deep learning-based semantic segmentation network. The proposed method, using the data of 69 participants (33 patients with MDD and 36 normal controls), achieved an average precision, recall, and dice similarity coefficient of 0.869, 0.865, and 0.852, respectively, in the automated segmentation task. Moreover, the intra-class correlation coefficient reached 0.870 in the volume estimation task. This study demonstrates that this deep learning-based method can provide accurate and quantitative analytic results of the habenula. By providing rapid and quantitative information on the habenula, we expect our proposed method will aid future psychiatric disease studies.

## Introduction

The habenula (Hb) is a paired epithalamic structure adjacent to the dorsomedial thalamus and the third ventricle^1^ that can be divided into distinct portions via different cellular morphological features. It integrates information received from the cerebral and limbic cortex and provides forebrain control over the activity of ascending monoaminergic projections from the brainstem^2^. Additionally, based on previous studies of Hb function, the Hb is involved in the pathogenesis of psychiatric disorders such as major depressive disorder (MDD)^3,4^. Compared to normal controls (NCs), the Hb volume of patients with MDD showed atrophy in a post-mortem study^5^.

According to previous post-mortem and structural imaging studies, the average volume of the human Hb is 15–30 mm^3^^5,6^. Several studies have reported comparing the volume of the Hb between patients with a psychiatric disorder and NCs: volume comparison among patients with different stages of MDD and NCs^7^; among medicated and unmedicated MDD patients, bipolar disorder patients, and NCs^8^; and among medicated and unmedicated patients with MDD and NCs^9^. The majority of previous human Hb volumetric studies have used manual segmentation to determine Hb volumes^7–10^. However, these conventional manual-based approaches are time-consuming and laborious, particularly with extensive datasets, and it is challenging to accurately produce the segmented masks due to the anatomical characteristics of the Hb. Thus, manual segmentation results of the Hb by different observers have large deviations and it is difficult to determine which fit the gold standard. To overcome this problem, two examiners trace the individual region and the reliability of their results are evaluated with an intra-class correlation^11^. Yet, this method is still time-consuming for the tracers. Overall, accurate Hb segmentation for quantitative analysis is still a challenging task. An accurate and quick Hb segmentation method might be a fundamental step in medical treatment, such as deep brain stimulation and neurosurgery, for targeting Hb sub-regions related to psychiatric diseases in the future^12,13^. For this reason, a couple of semi- or fully-automatic Hb segmentation approaches have been reported: (1) reproducibility of a myelin content-based Hb segmentation from 3 T magnetic resonance imaging (MRI) using a semi-automatic myelin contrast-based method^14^, and (2) a machine learning algorithm for fully-automatic Hb segmentation of 1.5 T MRI for Hb volume comparison of patients with bipolar disorder and schizophrenia with healthy controls^15^. Since those studies performed image processing such as intensity-based threshold and image registration^14,15^, there remain limitations in their ability to reliably perform automatic Hb segmentation in large MRI datasets. Accordingly, the development of accurate methods for an automated Hb segmentation of 7 T MRI in patients with depressive disorder is necessary. However, research on automatic analytic methods using a deep learning approach in the depressive disorder research field is currently scarce.

Recently, demonstrated as a powerful tool for semantic segmentation, deep learning methods based on convolutional neural networks can accommodate large annotated datasets and computational resources compared with traditional segmentation techniques^16,17^. Moreover, various studies have reported regional segmentation of the human brain and their performance using u-net-based semantic segmentation networks^18,19^. Nevertheless, there are no such reported cases of deep learning approaches for automated Hb segmentation. Thus, we developed a deep learning-based method for automated Hb segmentation using high-resolution 7 T MRI and assessed the clinical utility of this method using brain images of patients with MDD and NCs for the validation of our deep learning approach.

Although 7 T MRI is an imaging technique suitable for visualizing the Hb, it is still challenging to segment the Hb accurately using naked eye-based manual segmentation because of its low anatomical contrast and tiny size, resulting in low reliability of segmentation results from different observers. To address this limitation, we designed deep learning networks trained on manual segmentation masks from two different examiners. The final Hb segmentation results fused the two pre-trained networks’ outputs, taking into account both examiners’ manually segmented masks.

Additionally, to perform automatic anatomical structure segmentation, it is more efficient to focus on specific areas of the visual scene, picking out only important features of interest, similar to human visual attention, than to examine every part of the brain. The attention u-net was designed for this purpose and has been proposed to simply and accurately segment the pancreas, which occupies a small area in the abdomen^20,21^. In this study, therefore, we designed our deep learning networks’ architecture based on the attention u-net for robust and accurate Hb segmentation.

This study aimed to validate the reproducibility of our deep learning-based computer-aided tool via evaluating the automatic Hb segmentation performance and comparing manual and automated Hb volume estimation in individuals with MDD and NCs.

## Methods

### Study population

Patients with MDD and NCs ranging from 20 to 65 years of age were recruited from the psychiatric department of Gil Medical Center, Incheon, South Korea. At the screening evaluation, board-certified psychiatrists had structured interviews with the participants to assess their eligibility using the standard diagnostic instrument based on the Diagnostic and Statistical Manual of Mental Disorders, 5th edition (DSM-5)^22^. The severity of psychiatric symptoms of all participants was measured using the Hamilton Depression Rating Scale 17 items (HDRS-17)^23^, Beck Depression Inventory (BDI)^24^, and Clinical Global Impression Scale (CGI)^25,26^. This study was conducted in accordance with the Declaration of Helsinki and approved by the Institutional Review Board of the Gil Medical Center (IRB No. GDIRB2018-005), and written informed consent was obtained from all the participants.

The common eligibility criteria for the MDD and NC groups were as follows: (1) no previous abnormal findings on brain imaging; (2) no intellectual disability, neurocognitive disorders, or history of significant brain injury; (3) no personality disorder or substance use disorder including alcohol use disorder in the last year; (4) no major or unstable medical or neurological disorders in the last year; (5) no current serious suicide risk; (6) right-handedness using the Edinburgh Handedness Test; (7) not pregnant or lactating; and (8) no metal material in the body. The NCs were included according to the following additional criteria: (1) no family history of first-degree relatives with a major psychiatric disorder; (2) no history or symptoms of psychiatric disorders; (3) no history of taking psychotropics during their lifetime; and (4) a total score ≤ 6 on the HDRS-17. The participants who met the DSM-5 diagnostic criteria for MDD^27^ were included in the MDD group. The MDD and NC groups were matched for age and sex.

### Image acquisition

Whole-brain sagittal images were acquired using an 8-channel phased-array coil for 7-T MRI (MAGNETOM 7 T, Siemens, Erlangen, Germany). To evaluate the possibility of simultaneously recording relaxation times, such as T1 and T2*, the prototype multi-echo magnetization-prepared 2 rapid gradient echoes (ME-MP2RAGE) sequence by Siemens was utilized^28^. Image acquisition was performed using the following parameters: field of view (FOV) = 166 × 166 × 135.2 mm^3^ with a nominal isotropic resolution of 0.65 mm; matrix size = 256 × 256; 208 slices along the right-left axis (sagittal orientation); repetition time (TR) = 8000 ms; two inversion times (TIs) = 1000/3200 ms; flip angle (FA) = 4°; four echo times (TEs) = 3.46, 7.28, 11.1, and 14.92 ms; bandwidth = 280 Hz/px yielding an acquisition time (TA) = 14 min 16 s; bipolar readout; generalized auto-calibrating partially parallel acquisitions with acceleration factor = 3; and 7/8 and 6/8 partial Fourier factors along the phase-encoding and slice-encoding directions.

### Label acquisition

The manual segmentation was performed by two well-trained researchers using the T1 map of the participants’ 7 T MRIs. The researchers manually segmented the target voxels by tracing the Hb, which differed in signal intensity from that of the contiguous brain tissues, using three-dimensional analytic programs (i.e., ImageJ; ver. 1.52a). We used both medial and lateral parts for manual Hb delineation (generating gold-standard).

### Experimental overview

Two deep learning networks were trained for automatic Hb segmentation from the manual segmentation results of two different observers.

**Figure 1:**
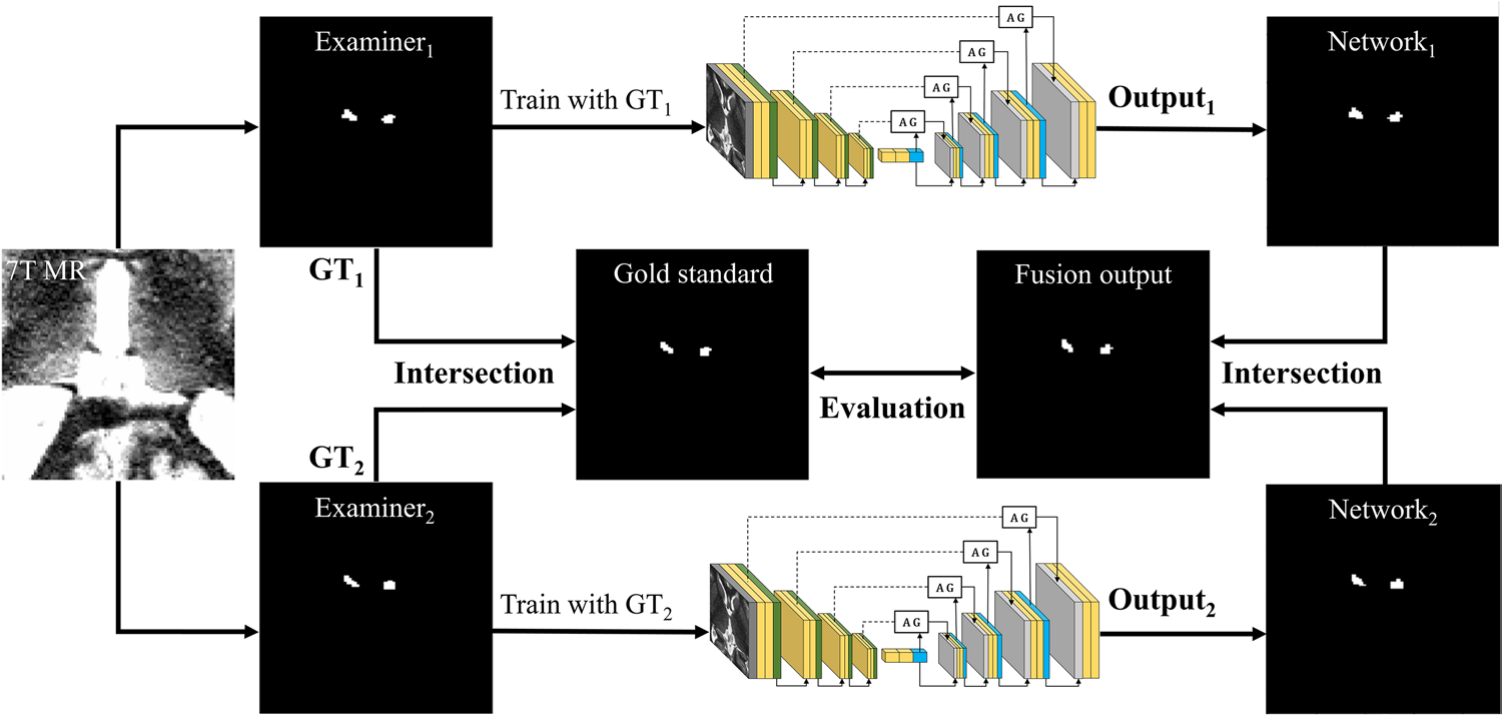
An illustrated overview of the automatic habenula segmentation. In the network training procedure, two manual segmentation masks were used for the training of two networks, and two segmentation results were obtained. The network evaluation was performed by comparing the intersected GT and fusion output. *GT* ground truth; *MR* magnetic resonance; *7 T* 7 Tesla; *AG* attention gate.

Figure 1 shows an overview of the network training and evaluation procedure for the automated Hb segmentation. $GT_{1}$ and $GT_{2}$ were obtained from two examiners using manual Hb segmentation. $GT_{1}$ was used to learn as the ground truth (GT) for $Network_{1}$ and $GT_{2}$ for $Network_{2}$. The final automatic segmented region of the Hb was the intersection of the automatic segmentation results from two different unfamiliar networks in the test dataset. To evaluate the automatic segmentation results, the fusion of different network outputs was compared with the gold standard as presented in the *Label acquisition* subsection.

### Preprocessing and experimental setup

**Figure 2:**
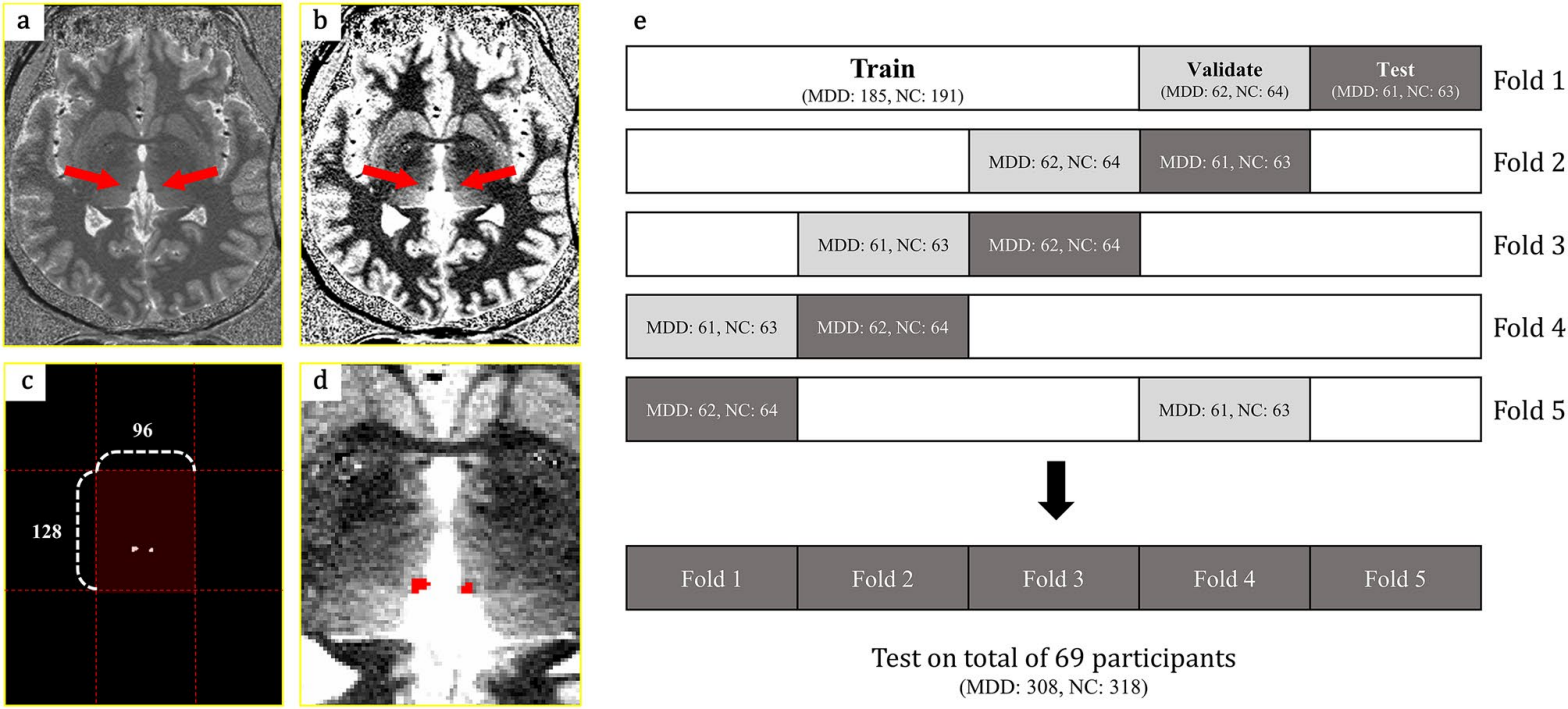
Preprocessing procedure of 7 T MRI (**a–d**), and experimental setup (**e**). (**a**) Original axial view 7 T MRI. (**b**) Window level (WL) and window width (WW) were set (WL/WW: 1300/750) to observe the Hb. (**c–d**) The region that included the Hb was coarsely chopped to the specific size. (**e**) An illustration of the data splitting method to conduct fivefold cross validation. *MRI* magnetic resonance image; *7 T* 7 Tesla; *Hb* habenula; *MDD* major depressive disorder; *NC* normal control.

We acquired a region of interest in the axial plane of the 7 T MRI volume (Fig. 2a). The window level and window width were set to clearly observe the Hb on 7 T MRI (window level: 1300, window width: 750) (Fig. 2b). To remove unnecessary brain regions, the images were uniformly cropped to 96 pixels (x-axis) and 128 pixels (y-axis), including the Hb (Fig. 2c,d). In this study, we excluded slices without the Hb to avoid a class imbalance issue. To train the segmentation network, we approximately divided the total brain MRI data (n = 69, 626 axial plane slices) by a ratio of 6: 2: 2 (train: validation: test). The whole dataset consisted of NC and MDD participants that were balanced across all folds (Fig. 2e). The range of the number of axial slices that included the Hb region for individual participants was 7–11 (min–max) slices.

### Network

**Figure 3:**
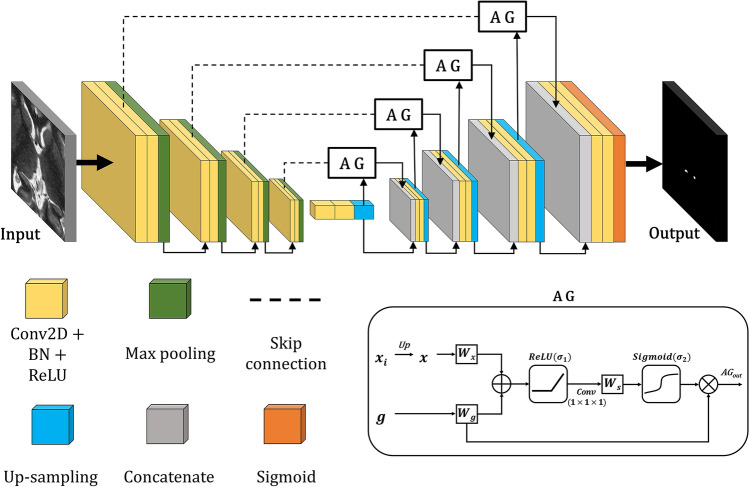
The architecture of the attention gate-based u-net for Hb segmentation. Each colored block including the AG process is indicated below the illustration of the network architecture. The significant feature maps were aggregated by the listed convolution operations, including AG, to segment the Hb regions. *Conv* convolution; *BN* batch normalization; *AG* attention gate; *ReLU* rectified linear unit; $x$: the feature maps of the previous layer; $g$: the skip connected feature maps; $W$: aggregation procedure of more than one feature with convolutional filters; $W_{s}$: aggregation procedure of only one feature with a single convolutional filter.

As shown in Fig. 3, we employed an attention u-net for Hb segmentation, which was obtained from the source codes (https://github.com/lixiaolei1982/Keras-Implementation-of-U-Net-R2U-Net-Attention-U-Net-Attention-R2U-Net.-). The network has an encoder path followed by a decoder path, each with four resolution steps (4 depths). Each decoder or encoder path contains two convolutional blocks (2 widths). The number of filters for feature aggregation increases to 64, 128, 256, or 512 depending on the depth of the network.

When the 7 T MRI was fed into the model, the significant feature maps were aggregated for Hb segmentation by the convolution operation. In the feature aggregation process, the feature map, which was reduced in resolution by a repeated pooling operation, was restored to the input image resolution by the up-sampling operation. Up-sampling was performed after the AG operation. The feed-forward procedure of the AG-based up-sampling was as follows:

$$AG_{{in}} = \sigma _{1} \left( {W_{\alpha } \left( x \right) + W_{\alpha } \left( g \right)} \right)$$

$$AG_{{out}} = \sigma _{2} \left( {W_{s} (AG_{{in}} } \right)) \times W_{\alpha } \left( g \right)$$

$$Concatenate = merge(AG_{{out}} ,~x)$$

where $\sigma _{1}$ is the rectified linear unit (ReLU) activation function, $\sigma _{2}$ is the sigmoid activation function, $W_{\alpha } \left( f \right)$ is a convolutional operation that maintains the number of feature map channels of $f$, and $W_{s} \left( f \right)$ is a convolutional operation that aggregates a single-channel from the $f$. The two input features maps, up-sampled feature map $x$ and skip-connected feature map ($g$), are added, after a convolution operation $W_{\alpha }$. After summation, the added feature map $W_{\alpha } \left( x \right) + W_{\alpha } \left( g \right)$ is activated by ReLU. The single feature map aggregated by $W_{s}$ is output as an activation map via sigmoid activation. The output of AG is a self-weighted feature map created by multiplying the activation map $\sigma _{2} \left( {W_{s} (AG_{{in}} } \right))$ and the skip-connected feature map $W_{\alpha } \left( g \right)$. Finally, the concatenate layer is a self-weighted feature map that focuses on the important features for segmentation of the Hb. The concatenate layer, including AG, affects the final network’s output by continuing to participate in the subsequent up-sampling operation.

### Implementation details

We trained our networks on a single Tesla V100 (32 GB) GPU (graphics processing unit). Each network consisted of 1,984,565 parameters. Our networks were trained using the Adam optimizer^29^ to jointly minimize the generalized dice loss^30^. We conducted early termination of the training procedure when loss did not improve during 50 epochs. The initial learning rate (LR) was 0.001, and when the loss did not minimize for 10 epochs, the LR was reduced by a factor of 0.2. The networks early terminated the training procedure in 100–300 epochs. In this study, we employed the following frameworks on Python (ver. 3.6.12): (1) Keras (ver. 2.2.5), TensorFlow-GPU (ver. 1.15.4) for deep learning analysis; and (2) Simple-ITK (ver. 1.2.0) for MRI preprocessing.

### Statistics

Demographic data and clinical characteristics were calculated and compared using two-tailed independent t-tests and chi-square tests. The software IBM SPSS Statistics (ver. 21.0) was used and *P* < 0.05 was set as the limit for statistical significance for these analyses.

We obtained the precision, recall, and dice similarity coefficient (DSC) by comparing the GT and automatic segmentation result of networks for evaluation in the test set.

To evaluate our network, we calculated the coincidence-rate of the GTs and auto-segmentation results. The evaluation was conducted voxel-wisely using the following equations:

$$Precision = \frac{{TP}}{{TP + FP}}$$

$$Recall~ = \frac{{TP}}{{TP + FN}}$$

$$DSC = \frac{{2 \times Precision \times Recall}}{{Precision + Recall}}$$

The true positive (TP), false positive (FP), true negative (TN), and false negative (FN) were obtained by comparing the voxels of a GT and fusion segmentation result. Since the whole dataset was divided into fivefold as a test dataset, we were able to evaluate every slice in our whole dataset (69 participants). Moreover, since deep neural networks (DNNs) are dependent on the training and validation set, we designed the training, validation, and test dataset to fivefold for every test dataset in each fold.

For the validation of clinical applications such as volume analysis, we compared manual and automated Hb segmentation in participants with MDD and NCs. Therefore, it was necessary to estimate the size of the Hb volume via 3D volume reconstruction for each participant (see Supplementary Fig. S1). In addition, we divided the total volume of the Hb into the left and right hemispheres to analyze automatic segmentation performance on each side.

After 3D reconstruction of the Hb, the intra-class correlation coefficients (ICCs) were calculated from each pair of brain volumes using the automatic and manual segmentation methods. Before this analysis, the normalization of the Hb volumes was performed using total intracranial volume (ICV). The Hb volumes were divided by the ICV for each participant $\left( {\frac{{Hb~Volume}}{{ICV}} \times 100\% } \right)$ to adjust for individual differences in brain size. After obtaining the number of voxels in the mask, the ICV was calculated by multiplying by the resolution. To make one mask, each subject's gray matter, white matter, and CSF from the 3 T MRI were segmented using SPM12 (see http://www.fil.ion.ucl.ac.uk/spm) and then summed. To assess the inter-rater reliability (i.e., the degree of agreement between the Hb volumes by automatic and manual segmentation), the ICC method involving the absolute agreement mode, which is sensitive to the differences in the mean values of observations, was used^31,32^. The reliability ICC ($r_{{ICC}}$) values were interpreted according to Cicchetti’s guidelines^33^ as follows: < 0.40, poor; 0.40–0.59, fair; 0.60–0.74, good; and 0.75–1.00, excellent. SPSS was used for the ICC analysis.

## Results

### Demographics

Supplementary Table S1 shows the demographics of the participants in this study. The age and sex ratio did not significantly differ between the two groups. The years of education and depressive symptom severity measured using the HDRS-17, BDI, and CGI differed significantly between the two groups.

### Evaluation of habenula segmentation

The average total number of voxels with automated segmentation for the Hb out of all voxels ($256 \times 256 \times 208$) was 24.01 ± 6.42 mm^3^ (mean ± standard deviation), and in the case of manual segmentation it was 24.19 ± 6.10 mm^3^. We divided the Hb volume into the left and right hemispheres in the MDD and NC groups. The Hb volumes from manual and automated segmentation in both groups were as follows: (1) left Hb in NC, 12.40 ± 4.00 mm^3^ and 11.98 ± 4.06 mm^3^ (manual method and automated method); (2) right Hb in NC, 12.21 ± 3.57 mm^3^ and 11.79 ± 3.26 mm^3^; (3) left Hb in MDD, 12.52 ± 3.08 mm^3^ and 12.58 ± 3.34 mm^3^; (4) right Hb in MDD, 11.21 ± 3.16 mm^3^ and 11.69 ± 2.82 mm^3^. The volumes from automated segmentation tended to be underestimated in NC participants and overestimated in MDD participants when compared to the manually segmented volumes. In the Pearson’s correlations of the clinical variables with the Hb volumes of the MDD group, there were no significant correlations: (1) HDRS-17: *r* = 0.158, *p* = 0.381; (2) BDI: *r* = 0.065, *p* = 0.718; (3) CGI: *r* = − 0.037, *p* = 0.838.

In addition, the average T1 value was 105,050 ± 27,476 (mean $\pm$ standard deviation) for automated segmentation and 106,395 ± 26,433 for manual segmentation, which was not significantly different between the two groups (paired *t*-test: NC group, $p$ = 0.145; MDD group, $p$ = 0.578).

Table 1 shows the performance evaluation of the automated Hb segmentation. The performance of our network reached a mean precision, recall, and DSC of 0.869, 0.865, and 0.852, respectively, using fivefold cross validation. The evaluation results of individual networks for fusion segmentation output are presented in Supplementary Table S2. The performance of $Network_{1}$ using $GT_{1}$ as the reference image reached a mean precision, recall, and DSC of 0.848, 0.817, and 0.815, respectively. Furthermore, the performance of $Network_{2}$ reached a mean precision, recall, and DSC of 0.852, 0.825, and 0.818, respectively. We also trained a single attention u-net from the intersected GT of the two raters for comparison with the proposed network. It achieved a mean precision, recall, and DSC of 0.847, 0.789, and 0.790, respectively. The proposed network achieved a higher recall than did the network that did not consider the two raters’ manual segmentation results (see Supplementary Table S3 & Fig. S2).

**Table 1:** The performance evaluation of the automatic segmentation results using the intersection network.

	Precision	Recall	DSC
**NC participants (n = 36)**			
Left Hb	0.885 ± 0.112	0.853 ± 0.176	0.847 ± 0.145
Right Hb	0.889 ± 0.156	0.871 ± 0.152	0.862 ± 0.127
Total Hb	0.883 ± 0.120	0.862 ± 0.109	0.863 ± 0.079
**MDD participants (n = 33)**			
Left Hb	0.868 ± 0.190	0.872 ± 0.191	0.855 ± 0.162
Right Hb	0.819 ± 0.198	0.867 ± 0.188	0.813 ± 0.158
Total Hb	0.848 ± 0.127	0.866 ± 0.149	0.842 ± 0.120
**Total participants (n = 69)**			
Left Hb	0.877 ± 0.175	0.862 ± 0.170	0.860 ± 0.147
Right Hb	0.854 ± 0.176	0.868 ± 0.173	0.846 ± 0.136
Total Hb	0.869 ± 0.124	0.865 ± 0.134	0.852 ± 0.094

The evaluation results are presented as mean and standard deviation.

*Hb* habenula, *DSC* dice similarity coefficient.

**Figure 4:**
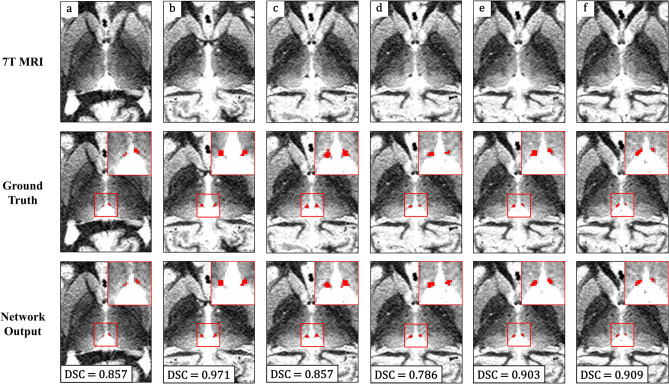
Segmentation results of several test cases (**a–f**). Presented MRIs were set to window level, 1300 and window width, 750 (top row). The ground truth is the intersection of two examiners (middle row). The prediction result is the intersection of the two trained segmentation networks (bottom row). Both ground truth and the automated segmentation results are presented as overlays on cross-sectional 7 T MRIs. *DSC* dice similarity coefficient; *MRI* magnetic resonance imaging; *7 T* 7 Tesla.

Figure 4 shows a cross-sectional view of the segmentation results of several cases. The segmentation performance of the automated segmentation model was highly consistent with GT. The cross validation results showed that the segmentation performance was similar for all folds, and there was a low standard deviation in each fold.

In addition, we conducted twofold cross validation to investigate the bias from using both MDD and NC data together for dataset generation. We separated total images into two folds: MDD participants for the training dataset and NC participants for the test dataset, and vice versa. Mann–Whitney U-tests showed significant differences in the mean precision (*p* < 0.001) and recall (*p* < 0.001) between the individual folds (Supplementary Table S4). In contrast, there was no significant difference (*p* = 0.283) between the mean DSCs from either fold.

### Intra-class correlation analysis

Table 2 shows the results of intra-class correlation analyses between automated and manual segmentation of the Hb volume. The mean of the normalized total Hb volume with automatic segmentation was 16.66 × 10^–4^ ± 4.39 × 10^–4^%, and the mean of the normalized total Hb volume with manual segmentation was 16.77 × 10^–4^ ± 4.05 × 10^–4^%. The ICCs calculating the agreement between the Hb volumes by automatic and manual segmentation were excellent for the MDD group ($r_{{ICC}}$ = 0.818, $p$ < 0.001, n = 33), NC group ($r_{{ICC}}$ = 0.897, $p$ < 0.001, n = 36), and participants overall ($r_{{ICC}}$ = 0.870, $p$ < 0.001, n = 69).

**Table 2:** The intra-class correlation analysis between automatic and manually segmented habenula volumes.

	Habenula volume^a^		Intra-class correlation	
	Automatic estimation	Manual estimation	ICC	*p*-value
**NC (n = 36)**				
Left Hb	8.11 × 10^–4^ ± 2.91 × 10^–4^	8.55 × 10^–4^ ± 2.79 × 10^–4^	0.903	**< 0.001**
Right Hb	7.89 × 10^–4^ ± 2.37 × 10^–4^	8.30 × 10^–4^ ± 2.31 × 10^–4^	0.819	**< 0.001**
Total Hb	16.00 × 10^–4^ ± 5.04 × 10^–4^	16.85 × 10^–4^ ± 4.52 × 10^–4^	0.897	**< 0.001**
**MDD (n = 33)**				
Left Hb	8.93 × 10^–4^ ± 2.05 × 10^–4^	8.72 × 10^–4^ ± 1.99 × 10^–4^	0.920	**< 0.001**
Right Hb	8.45 × 10^–4^ ± 1.88 × 10^–4^	7.96 × 10^–4^ ± 2.31 × 10^–4^	0.658	**0.001**
Total Hb	17.38 × 10^–4^ ± 3.49 × 10^–4^	16.68 × 10^–4^ ± 3.54 × 10^–4^	0.818	**< 0.001**
**Total participants (n = 69)**				
Left Hb	8.50 × 10^–4^ ± 2.55 × 10^–4^	8.63 × 10^–4^ ± 2.42 × 10^–4^	0.908	**< 0.001**
Right Hb	8.16 × 10^–4^ ± 2.15 × 10^–4^	8.14 × 10^–4^ ± 2.30 × 10^–4^	0.750	**< 0.001**
Total Hb	16.66 × 10^–4^ ± 4.39 × 10^–4^	16.77 × 10^–4^ ± 4.05 × 10^–4^	0.870	**< 0.001**

Significant results are indicated in bold.

*SD* standard deviation, *ICC* intra-class correlation coefficient.

^a^Habenula volumes were normalized using total intracranial volume (ICV). Habenula volumes were divided by the ICV for each participant as a normalization process (regional brain volume/ ICV × 100%) for the subsequent analysis. Normalized habenula volumes are described as mean ± SD.

**Figure 5:**
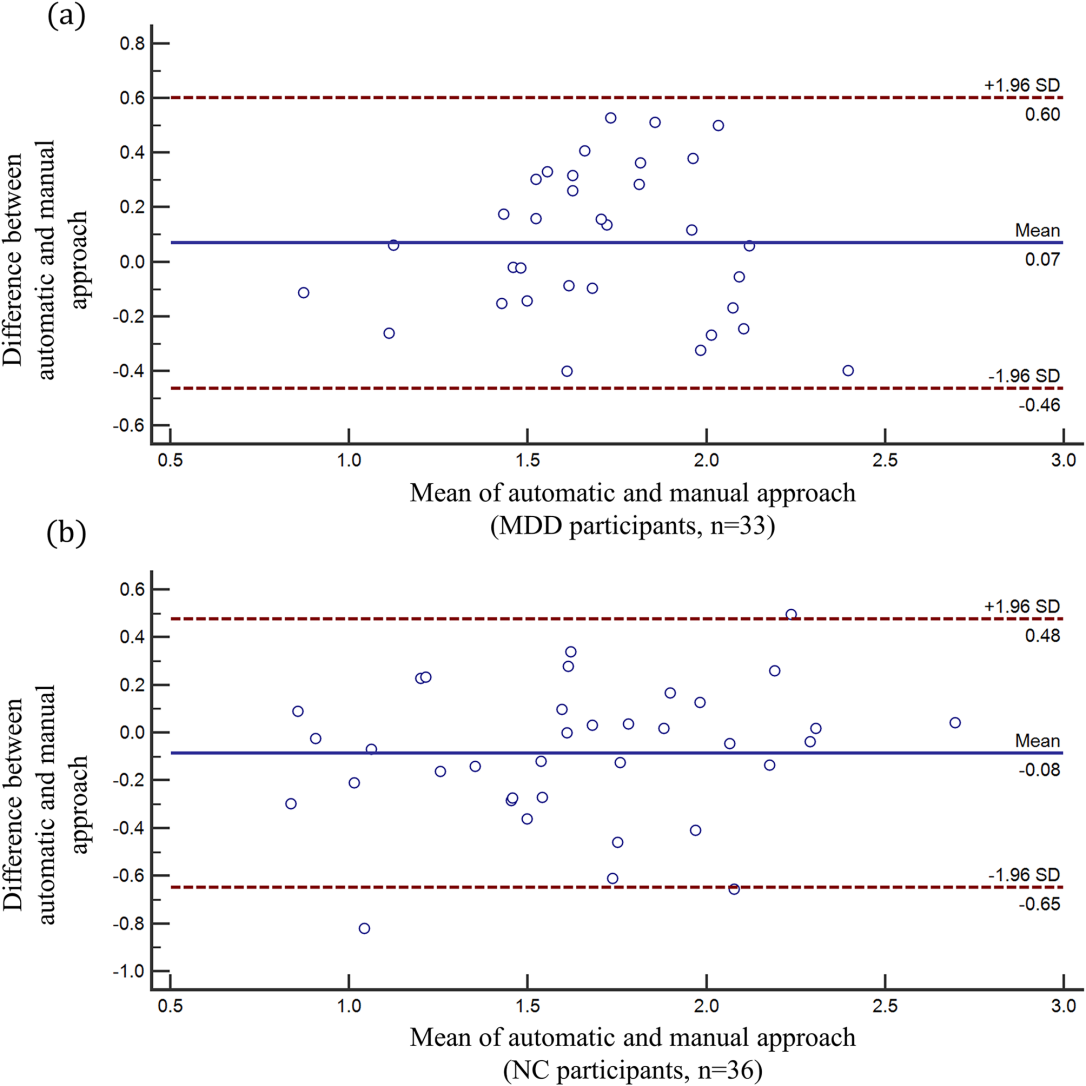
Bland–Altman analysis assessing the reproducibility of the automatic habenula segmentation method for (**a**) MDD participants and (**b**) NC participants. Habenula volumes were divided by the intracranial volume (ICV) for each participant (regional brain volume/ ICV × 100%) as a normalization process for the subsequent analysis. *MDD *major depressive disorder; *NC* normal control; *SD* standard deviation.

Significant ICCs were obtained for both the left and right Hb. In the left Hb, excellent ICCs were observed in the MDD group ($r_{{ICC}}$ = 0.920, $p$ < 0.001), NC group ($r_{{ICC}}$ = 0.903, $p$ < 0.001), and participants overall ($r_{{ICC~}}$ = 0.908, $p$ < 0.001). In the right Hb, we obtained excellent ICC values in the NC group ($r_{{ICC~}}$ = 0.819, $p$ < 0.001) and participants overall ($r_{{ICC}}$ = 0.750, $p$ < 0.001), and a good ICC value for the MDD group ($r_{{ICC}}$ = 0.658, $p$ = 0.001). The Bland–Altman analyses of the estimated Hb volumes from automatic and manual segmentation in the MDD and NC groups are presented in Fig. 5. The Bland–Altman plots show the reproducibility of the automatic Hb segmentation method for Hb volume estimation.

## Discussion

In this study, we proposed a deep attention u-net-based intersection network for accurate Hb segmentation and quantitative Hb analysis. As a result of experiments, the mean precision, recall, and DSC in the automatic segmentation using the intersection of attention u-net was good in the total participants. Additionally, the ICCs between automatic and manual segmentation of the total Hb were excellent in all participants, participants with MDD, and NCs. Therefore, we suggest that the proposed approach is suitable for the segmentation of the Hb, which is a brain region tiny in size with low contrast in brain MRI. To the best of our knowledge, this is the first study which presented an automatic Hb segmentation based on volume estimation method in participants with MDD and NCs using 7 T MRI.

In the automatic segmentation procedure, we obtained a mean precision, recall, and DSC of 0.869, 0.865, and 0.852, respectively. In recent years, a couple of studies on automated segmentation of the Hb have been reported. The first study performed semi- and fully-automated segmentation in 3 T MRI of healthy young adults^14^, and the second study performed fully-automated segmentation in children, adolescents, and adults with bipolar disorder and schizophrenia^15^. In the first study, the DSC for binary segmentation reached 0.71 for semi-automated segmentation and 0.69 for fully-automated segmentation, and the DSC of the probability map reached 0.74 for both semi- and fully-automated segmentation^14^. In a more recent study that segmented the Hb with a fully-automated framework, the DSC of the inter-rater reliability tests between manual and automatic segmentation ranged between 0.758 and 0.828^15^. Although the participants in the previous studies had different clinical characteristics from those in our study, our automatic Hb segmentation approach seems to be more accurate (mean DSC > 0.85) than that of the other studies.

In this study, the $r_{{ICC}}$ of the total Hb ranged between 0.818 and 0.897, depending on the group. In a previous study conducted on healthy young adults, the $r_{{ICC}}$ for the Hb was 0.62 for semi-automated segmentation and 0.47 for fully-automated segmentation^14^, which shows the superiority of our approach. However, the $r_{{ICC}}$ of the Hb was different between the groups and hemispheres in our study. According to the ICC analysis, the $r_{{ICC}}$ of the left Hb was excellent (0.903–0.920), while the $r_{{ICC}} ~$ of the right Hb was from 0.658 (MDD) to 0.819 (NC). Specifically, the volume estimation performance for the right Hb of MDD participants was slightly lower than that of NC participants. With respect to the right Hb, the volume in some of the MDD participants was low, which might be one reason for the discrepancy between the NC and MDD groups, both in the Hb segmentation and in the volume estimation. In addition, the asymmetry of the left and right Hb also might be a reason^34^.

Another attribute of our approach is that the results of two networks, each trained on GT generated by two different observers, were intersected to output fusion segmentation results. When trained with the intersected GT, the network reached a low mean recall (0.789) compared to that of the single attention-network (0.865). Since the Hb is a very small region in the brain, there may be slight differences in the manual delineation results from two independent raters. Accordingly, we assume that training and intersecting independent networks according to individual raters (i.e., $GT_{1}$ and $GT_{2}$) would achieve more reliable segmentation results than a single network that encoded both raters (e.g., union or intersection of GTs).

Our approach is different from the previous studies for following reasons: first, this is the first automated segmentation study performed in participants with MDD and NCs using high-resolution 7 T MRI that can ideally visualize the Hb. Second, a DNNs approach for automatic Hb segmentation and volume estimation was conducted. We designed a deep learning network based on the attention u-net that was optimized for segmenting small objects (i.e., the Hb) of various shapes. Third, since the segmentation was performed by the fusion of the two pre-trained attention u-net using two different GTs, it is believed that a more reliable segmentation was achieved.

We investigated whether there was bias in our network training procedure that included both MDD and NC participants. There were two limitations in this bias check process: (1) an imbalance in the number of images in the training and test sets, and (2) a decrease in the number of images in the training set. However, for the bias check, we divided the total images into an MDD dataset and an NC dataset (MDD, 308 axial slices; NC, 318 axial slices), assessing the results when using one dataset for training and the other for testing. We obtained significantly different results in the mean precision and recall, but not DSC, between the individual folds. However, further study is necessary to investigate potential bias using a large 7 T MRI dataset.

The high DSC and reproducibility of the automated segmentation of this study demonstrate that the applicability of the DNNs’ approach for Hb volume estimation in 7 T MRI is promising. Although the Hb is considered to be an important brain region in the etiology of major psychiatric disorders, its small size has made it difficult to investigate via neuroimaging. The Hb is involved in emotional and cognitive processes, having connections to many other areas of the brain (e.g., thalamus, prefrontal cortex, basal ganglia, and brainstem monoaminergic neurotransmitter systems)^35,36^. Recently, there are many studies focused on the connectivity between the Hb and other brain regions of interest such as monoamine centers and the thalamus in depression^37,38^. However, manual segmentation is time-consuming, highly variable, and the rater must acquire a high level of technical ability and anatomical knowledge for accurate segmentation, which has become a significant barrier to entry into this field of research^15^.

Considering that the data acquired through neuroimaging research is gradually increasing and that machine learning techniques are becoming more popular, the automatic segmentation approach in our study is expected to be a useful tool for many future studies.

## Conclusion

This study presented an intersection network based on attention u-net for an automated segmentation of the Hb using 7 T MRI that performed automatic segmentation and estimated the Hb volume with high accuracy and reproducibility (i.e., high DSC and correlation coefficients). Furthermore, it is expected that the proposed automatic Hb segmentation method will be useful for future psychiatric neuroimaging studies to facilitate automatic segmentation and volume estimation of the Hb and other important small brain regions in 7 T MRI.

## Supplementary Information

Supplementary Information.

## Data Availability

The datasets generated during or analyzed during the current study are available from the corresponding author on reasonable request.

## References

[1] Hikosaka O, Sesack SR, Lecourtire L, Shepard PD (2008). Habenula: crossroad between the basal ganglia and the limbic system. J. Neurosci..

[2] Poller WC (2011). Lateral habenular neurons projecting to reward-processing monoaminergic nuclei express hyperpolarization-activated cyclic nucleotid-gated cation channels. Neuroscience.

[3] Batalla A (2017). The role of the habenula in the transition from reward to misery in substance use and mood disorders. Neurosci. Biobehav. Rev..

[4] Lecca S, Meye FJ, Mameli M (2014). The lateral habenula in addiction and depression: An anatomical, synaptic and behavioral overview. Eur. J. Neurosci..

[5] Ranft K (2009). Evidence for structural abnormalities of the human habenular complex in affective disorders but not in schizophrenia. Psychol. Med..

[6] Savitz JB, Rauch SL, Drevets WC (2013). Reproduced from Habenula volume in bipolar disorder and major depressive disorder: A high-resolution magnetic resonance imaging study. Mol. Psychiatry..

[7] Carceller-Sindreu M (2015). Volumetric MRI study of the habenula in first episode, recurrent and chronic major depression. Eur. Neuropsychopharmacol..

[8] Savitz JB (2011). Habenula volume in bipolar disorder and major depressive disorder: A high-resolution magnetic resonance imaging study. Biol. Psychiatry..

[9] Schmidt FM (2017). Habenula volume increases with disease severity in unmedicated major depressive disorder as revealed by 7T MRI. Eur. Arch. Psychiatry Clin. Neurosci..

[10] Lawson RP, Drevets WC, Roiser JP (2013). Defining the habenula in human neuroimaging studies. Neuroimage.

[11] Pantel J (2000). A new method for the in vivo volumetric measurement of the human hippocampus with high neuroanatomical accuracy. Hippocampus.

[12] Akram H (2017). Subthalamic deep brain stimulation sweet spots and hyperdirect cortical connectivity in Parkinson's disease. Neuroimage.

[13] Morris LS (2019). Ultra-high field MRI reveals mood-related circuit disturbances in depression: A comparison between 3-Tesla and 7-Tesla. Transl. Psychiatry.

[14] Kim JW (2018). Reproducibility of myelin content-based human habenula segmentation at 3 Tesla. Hum. Brain Mapp..

[15] Germann J (2020). Fully automated habenula segmentation provides robust and reliable volume estimation across large MRI datasets suggesting intriguing developmental trajectories in psychiatric disease. Biol. Psychiatry Cogn. Neurosci. Neuroimaging..

[16] Shelhamer E, Long J, Darrell T (2017). Fully convolutional networks for semantic segmentation. IEEE Trans. Pattern Anal. Mach. Intell..

[17] LeCun, Y. & Bengio, Y. *Convolutional networks for images, speech, and time series*.(ed. Arbib, M.A.). 255–258 (MIT press, 1995).

[18] Nguyen, D.M.H., Vu, H.T., Ung, H.Q. & Nguyen, G.T. 3D-brain segmentation using deep neural network and gaussian mixture model. in *proceedings of the IEEE Winter Conference on Applications of Computer Vision*. 815–824 (IEEE, 2017).

[19] Ding, Z., Han, X. & Niethammer, M. Votenet +: An Improved Deep Learning Label Fusion Method for Multi-Atlas Segmentation. In *proceedings of 2020 IEEE International Symposium on Biomedical Imaging*. 363–367 (IEEE, 2020). 10.1109/isbi45749.2020.9098493 PMC8899817 35261721

[20] Oktay, O., *et al*. Attention u-net: learning where to look for the pancreas. In *proceedings of the Conference on Medical Imaging with Deep Learning*. (MIDL, 2018).

[21] Anderson, P., *et al*. Bottom-up and top-down attention for image captioning and visual question answering. In *Proceedings of the IEEE conference on computer vision and pattern recognition*. 6077–6086 (IEEE, 2018).

[22] First, M., *et al*. *Structured Clinical Interview for DSM-5-Research Version (SCID-5 for DSM-5, Research Version; SCID-5-RV)* 1–94 (American Psychiatric Association , 2015).

[23] Yi JS (2005). Validity and reliability of the Korean version of the Hamilton Depression Rating Scale (K-HDRS). J. Korean Neuropsychiatr. Assoc..

[24] Han HM (1986). Korean standardization study of Beck Depression Inventory in Korea. J. Korean Neuropsychiatric Assoc..

[25] Busner J, Targum SD (2007). The clinical global impressions scale: Applying a research tool in clinical practice. Psychiatry.

[26] Haro JM (2003). The Clinical Global Impression-Schizophrenia scale: A simple instrument to measure the diversity of symptoms present in schizophrenia. Acta Psychiatr. Scand. Suppl..

[27] American Psychiatric Association. *Diagnostic and statistical manual of mental disorders (DSM-5®)* (American Psychiatric Association, 2013).

[28] Metere R, Kover T, Möller HE, Schäfer A (2017). Simultaneous quantitative MRI mapping of T1, T2* and magnetic susceptibility with multi-echo MP2RAGE. PLoS ONE.

[29] Kingma, D.P. & Ba, J. Adam: A method for stochastic optimization. In *proceedings of the International Conference on Learning Representations*. (ICLR, 2014).

[30] Sudre, C.H., Li, W., Vercauteren, T., Ourselin, S. & Cardoso, M. Generalised dice overlap as a deep learning loss function for highly unbalanced segmentation. in *proceeding of the International Workshop on Deep Learning in Medical Image Analysis*. **10553**, 240–248 (Springer, 2017). 10.1007/978-3-319-67558-9_28 PMC7610921 34104926

[31] Fisher, R.A. *Statistical Methods, Experimental Design, and Scientific Inference* (ed. Bennett J.H.) (Oxford University Press, 1990).

[32] Fisher, R.A. *Statistical Methods for Research Workers*. 356 (Oliver and Boyd, 1954).

[33] Cicchetti DV (1994). Guidelines, criteria, and rules of thumb for evaluating normed and standardized assessment instruments in psychology. Psychol. Assess..

[34] Ahumada-Galleguillos P (2017). Directional asymmetry in the volume of the human habenula. Brain Struct. Funct..

[35] Benarroch EE (2015). Habenula: Recently recognized functions and potential clinical relevance. Neurology.

[36] Boulos LJ, Darcq E, Kieffer BL (2017). Translating the habenula-from rodents to humans. Biol Psychiatry..

[37] Ely BA (2016). Resting-state functional connectivity of the human habenula in healthy individuals: Associations with subclinical depression. Hum. Brain Mapp..

[38] Luan SX, Zhang L, Wang R, Zhao H, Liu C (2019). A resting-state study of volumetric and functional connectivity of the habenular nucleus in treatment-resistant depression patients. Brain Behav..

