# Supplementary material for: Reproducibility of automated habenula segmentation via deep learning in major depressive disorder and normal controls with 7 Tesla MRI
Source: Sci Rep. 2021 Jun 29;11:13445. doi: 10.1038/s41598-021-92952-z (PMC8241874; doi:10.1038/s41598-021-92952-z)
Supplement: Supplementary file 1 — Supplementary Information. [file 41598_2021_92952_MOESM1_ESM.pdf]

Supplementary Information

Reproducibility of automated habenula segmentation via deep learning in major depressive disorder and normal controls with 7 Tesla MRI

Sang-Heon Lim[1,2,†], Jihyun Yoon[3,†], Young Jae Kim[2], Chang-Ki Kang[4], Seo-Eun Cho[5], Kwang Gi Kim[1,2,*] & Seung-Gul Kang[5,*]

[1]*Department of Health Sciences and Technology, Gachon Advanced Institute for Health Sciences and Technology (GAIHST), Gachon University, Seongnam-si, Republic of Korea;*

[2]*Department of Biomedical Engineering, College of Medicine, Gachon University, Seongnam-si, Republic of Korea;*

[3]*Department of Family Medicine, Yongin Severance Hospital, Yonsei University College of Medicine, Yongin, Republic of Korea;*

[4]*Department of Radiological Science, College of Health Science, Gachon University, Incheon, Republic of Korea;*

[5]*Department of Psychiatry, Gil Medical Center, Gachon University College of Medicine, Incheon, Republic of Korea;*

This PDF file includes:

Figures. S1 to S2

Tables. S1 to S4

Supplementary Tables

Supplementary Table S1. Demographic and clinical characteristics of the participants and their comparison between the MDD and normal control groups.

Properties	Total (n = 69)	MDD (n = 33)	NC (n = 36)	Statistics	
Age, years	37.7 ± 13.1	40.4 ± 14.1	34.7 ± 11.0	$t = 1.76$	$p = 0.084$
Sex (male:female)	20:49	8:25	12:24	$\chi^2 = 0.69$	$p = 0.406$
Education, years	14.0 ± 3.0	12.7 ± 3.4	15.2 ± 1.9	$t = –3.37$	$p = 0.001$
HDRS-17 score	8.8 ± 7.9	15.6 ± 5.8	2.5 ± 2.4	$t = 13.03$	$p < 0.001$
BDI score	15.1 ± 15.2	27.8 ± 12.7	3.5 ± 3.7	$t = 10.62$	$p < 0.001$
CGI score	2.5 ± 1.7	4.0 ± 1.0	1.1 ± 0.2	$t = 17.88$	$p < 0.001$

Data are mean±SD or number

Abbreviations: MDD, major depressive disorder; SD, standard deviation; NC, normal control; HDRS-17, Hamilton Depression Rating Scale-17 items; BDI, Beck Depression Inventory; CGI, Clinical Global Impression Scale

Supplementary Table S2. Evaluation of individual networks for the intersection network.

	Precision	Recall	DSC
$Rater_1$			
Fold 1	0.867 ± 0.152	0.748 ± 0.223	0.778 ± 0.176
Fold 2	0.818 ± 0.197	0.795 ± 0.189	0.793 ± 0.167
Fold 3	0.879 ± 0.131	0.824 ± 0.133	0.836 ± 0.099
Fold 4	0.839 ± 0.125	0.823 ± 0.143	0.817 ± 0.095
Fold 5	0.817 ± 0.162	0.855 ± 0.146	0.817 ± 0.112
**Average**	**0.848 ± 0.145**	**0.817 ± 0.162**	**0.815 ± 0.120**
$Rater_2$			
Fold 1	0.839 ± 0.164	0.772 ± 0.214	0.778 ± 0.165
Fold 2	0.888 ± 0.127	0.771 ± 0.175	0.807 ± 0.125
Fold 3	0.888 ± 0.143	0.800 ± 0.172	0.817 ± 0.116
Fold 4	0.847 ± 0.153	0.851 ± 0.144	0.830 ± 0.113
Fold 5	0.853 ± 0.134	0.855 ± 0.146	0.841 ± 0.105
**Average**	**0.852 ± 0.149**	**0.825 ± 0.170**	**0.818 ± 0.125**

The evaluation results are presented as mean and standard deviation.

Abbreviations: DSC, dice similarity coefficient

Supplementary Table S3. The automatic segmentation results of a single attention-network trained with intersected ground truth.

	Precision	Recall	DSC
Fold 1	0.824 ± 0.046	0.750 ± 0.057	0.759 ± 0.202
Fold 2	0.852 ± 0.184	0.782 ± 0.182	0.798 ± 0.156
Fold 3	0.871 ± 0.155	0.808 ± 0.160	0.818 ± 0.123
Fold 4	0.833 ± 0.171	0.861 ± 0.150	0.831 ± 0.131
Fold 5	0.855 ± 0.164	0.744 ± 0.208	0.776 ± 0.164
Average	**0.847 ± 0.143**	**0.789 ± 0.150**	**0.790 ± 0.154**

The evaluation results are presented as mean and standard deviation.

Abbreviations: DSC, dice similarity coefficient

Supplementary Table S4. Two-fold cross validation metrics investigating potential bias in using only the MDD (n=33, 308 axial slices) or NC (n=36, 318 axial slices) dataset in training our network.

	Precision	Recall	DSC
Fold 1[a]	**0.815 ± 0.174**	**0.842 ± 0.176**	0.812 ± 0.148
Fold 2[b]	**0.882 ± 0.147**	**0.784 ± 0.167**	0.801 ± 0.126

Mann-Whitney U-tests were used to assess significant differences in the metrics from using the MDD or NC participants as the training dataset. We used a significance level of 0.05 and significantly different results are indicated in bold. The evaluation metrics are presented as mean ± standard deviation.

[a] Training data: MDD participants; test data: NC participants.

[b] Training data: NC participants; test data: MDD participants.

Abbreviations: DSC, dice similarity coefficient; MDD, major depressive disorder; NC, normal control.

Supplementary Figures

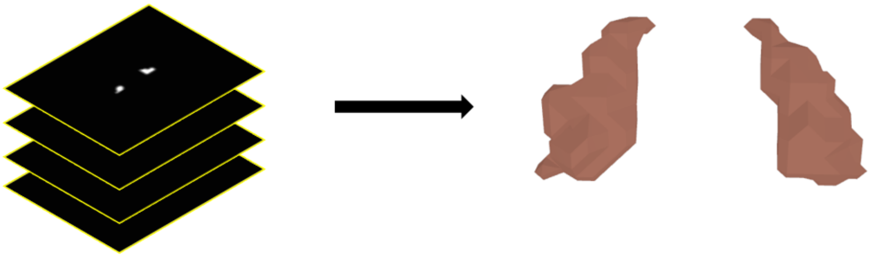

Supplementary Figure S1. An example of the 3D volume reconstruction for the habenula volume calculation.

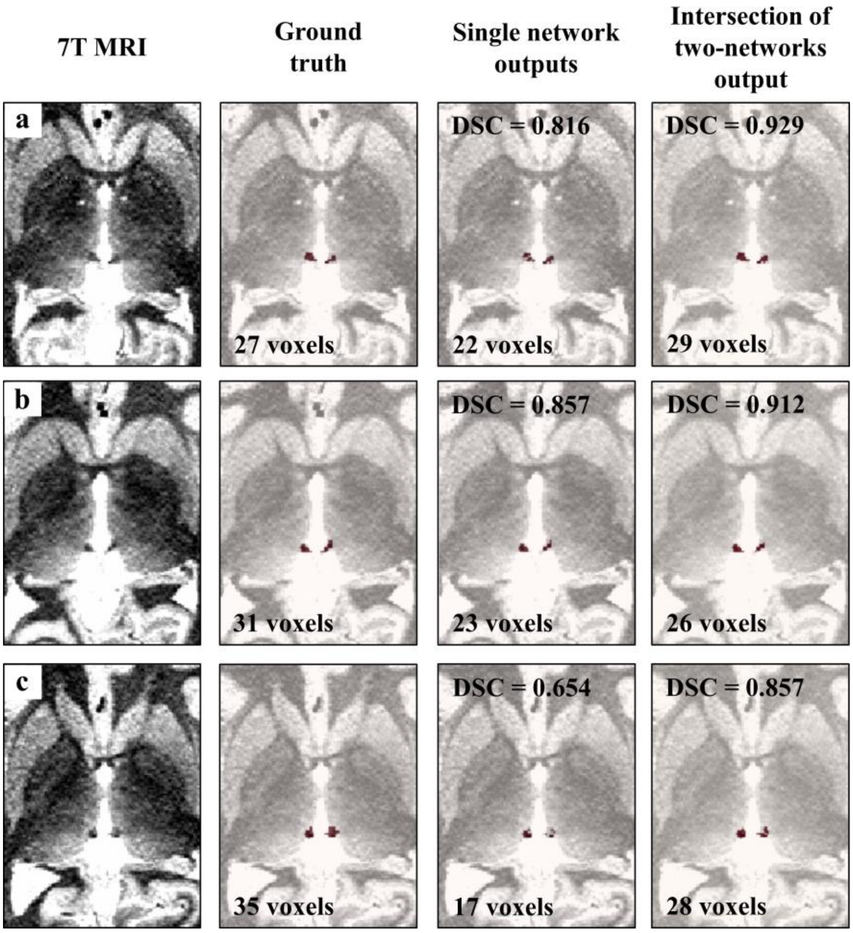

Supplementary Figure S2. Comparison of the two training methods.

From the left, 7 Tesla MRI, gold-standard data (ground truth), segmentation results of a single attention-network, and intersected segmentation results of a fusion attention-network. The single attention-network was trained with the intersected ground truths obtained from two different examiners. The proposed network is a parallel structure that trains two different ground truths separately. The fusion segmentation results were generated using the intersections of the segmentation results of two attention u-net networks. (**a–c**) Axial images from three different participants. Due to the low sensitivity of the single network, the DSCs and predicted region of voxels were lower than the proposed network.

Abbreviations: MRI, magnetic resonance imaging; DSC, dice similarity coefficient
